# Author Correction: PSIP1/LEDGF reduces R-loops at transcription sites to maintain genome integrity

**DOI:** 10.1038/s41467-024-49334-6

**Published:** 2024-06-07

**Authors:** Sundarraj Jayakumar, Manthan Patel, Fanny Boulet, Hadicha Aziz, Greg N. Brooke, Hemanth Tummala, Madapura M. Pradeepa

**Affiliations:** 1 Blizard Institute; Faculty of Medicine and Dentistry, Queen Mary University of London, London, UK; 2 Bhabha Atomic Research Centre, Mumbai, India; 3 School of Life Sciences, University of Essex, Colchester, UK

**Keywords:** Homologous recombination, Transcription, RNA decay

Correction to: *Nature Communications* 10.1038/s41467-023-44544-w, published online 08 January 2024

The original version of this Article contained an error in Fig. 1l, in which the labelling of S9.6 and PSIP1 in the genome browser shot is inadvertently swapped. This has now been corrected both in the html and pdf version.

