# Supplementary material for: PSIP1/LEDGF reduces R-loops at transcription sites to maintain genome integrity
Source: Nat Commun. 2024 Jan 8;15:361. doi: 10.1038/s41467-023-44544-w (PMC10774266; doi:10.1038/s41467-023-44544-w)
Supplement: Supplementary file 3 — Description of Additional Supplementary Files [file 41467_2023_44544_MOESM3_ESM.pdf]

**Description of Additional Supplementary Files**

File Name: Supplementary Data 1
Description: List of genes known to play role in R-loop regulation

File Name: Supplementary Data 2
Description: Reagents used in the study
