# Supplementary material for: PSIP1/LEDGF reduces R-loops at transcription sites to maintain genome integrity
Source: Nat Commun. 2024 Jan 8;15:361. doi: 10.1038/s41467-023-44544-w (PMC10774266; doi:10.1038/s41467-023-44544-w)
Supplement: Supplementary file 6 — Reporting Summary [file 41467_2023_44544_MOESM6_ESM.pdf]

Corresponding author(s): Pradeepa Madapura
Last updated by author(s): Dec 14, 2023

Reporting Summary

Nature Portfolio wishes to improve the reproducibility of the work that we publish. This form provides structure for consistency and transparency in reporting. For further information on Nature Portfolio policies, see our Editorial Policies and the Editorial Policy Checklist.

Statistics

For all statistical analyses, confirm that the following items are present in the figure legend, table legend, main text, or Methods section.

n/a	Confirmed	
☐	☒	The exact sample size (*n*) for each experimental group/condition, given as a discrete number and unit of measurement
☐	☒	A statement on whether measurements were taken from distinct samples or whether the same sample was measured repeatedly
☐	☒	The statistical test(s) used AND whether they are one- or two-sided<br>*Only common tests should be described solely by name; describe more complex techniques in the Methods section.*
☒	☐	A description of all covariates tested
☐	☒	A description of any assumptions or corrections, such as tests of normality and adjustment for multiple comparisons
☐	☒	A full description of the statistical parameters including central tendency (e.g. means) or other basic estimates (e.g. regression coefficient) AND variation (e.g. standard deviation) or associated estimates of uncertainty (e.g. confidence intervals)
☐	☒	For null hypothesis testing, the test statistic (e.g. *F*, *t*, *r*) with confidence intervals, effect sizes, degrees of freedom and *P* value noted<br>*Give P values as exact values whenever suitable.*
☒	☐	For Bayesian analysis, information on the choice of priors and Markov chain Monte Carlo settings
☒	☐	For hierarchical and complex designs, identification of the appropriate level for tests and full reporting of outcomes
☒	☐	Estimates of effect sizes (e.g. Cohen's *d*, Pearson's *r*), indicating how they were calculated

Our web collection on statistics for biologists contains articles on many of the points above.

Software and code

Policy information about availability of computer code

Data collection	No softwares was used for data collection
Data analysis	Bowtie2 http://bowtie-bio.sourceforge.net/bowtie2/ v2.4.5<br>STAR https://github.com/alexdobin/STAR v2.7.0f<br>SAMtools http://www.htslib.org/ v1.10<br>BEDTools https://github.com/arq5x/bedtools2/ v2.28.0<br>deepTools https://github.com/deeptools/deepTools v3.5.1<br>DESeq2 https://github.com/mikelove/DESeq2/ v1.34.0<br>Intervene https://github.com/asntech/intervene v0.6.5<br>SEACR https://github.com/FredHutch/SEACR v1.3<br>Trimmomatic v0.36<br>GraphPad Prsim v9

For manuscripts utilizing custom algorithms or software that are central to the research but not yet described in published literature, software must be made available to editors and reviewers. We strongly encourage code deposition in a community repository (e.g. GitHub). See the Nature Portfolio guidelines for submitting code & software for further information.

Data

Policy information about availability of data

All manuscripts must include a data availability statement. This statement should provide the following information, where applicable:

- Accession codes, unique identifiers, or web links for publicly available datasets
- A description of any restrictions on data availability
- For clinical datasets or third party data, please ensure that the statement adheres to our policy

All the NGS data generated in this study can be accessed at NBCI GEO under the accession ID GSE220234
CUT&Tag, RNAseq and TTseq datasets are deposited in the NCBI GEO datasets with following accession IDs.
CUT&Tag GSE220231
CUT&Tag (HEK293T) GSE245788
RNAseq GSE220232
TTseq GSE220233

Human research participants

Policy information about studies involving human research participants and Sex and Gender in Research.

Reporting on sex and gender	NA
Population characteristics	NA
Recruitment	NA
Ethics oversight	NA

Note that full information on the approval of the study protocol must also be provided in the manuscript.

Field-specific reporting

Please select the one below that is the best fit for your research. If you are not sure, read the appropriate sections before making your selection.

☒ Life sciences ☐ Behavioural & social sciences ☐ Ecological, evolutionary & environmental sciences

For a reference copy of the document with all sections, see nature.com/documents/nr-reporting-summary-flat.pdf

Life sciences study design

All studies must disclose on these points even when the disclosure is negative.

Sample size	As standard practice in the field, all the experiments were concluded based on independent biological repeats as mentioned in the figure legends
Data exclusions	No data was excluded in the study
Replication	Biological replicates were used to ensure data is reproducible
Randomization	Randomization for the samples was not required as a part of this study, as the experimentation was on cell lines .
Blinding	In some microscopy studies the sample were score blind. But in IP and immunoblotting blinding was not possible.

Reporting for specific materials, systems and methods

We require information from authors about some types of materials, experimental systems and methods used in many studies. Here, indicate whether each material, system or method listed is relevant to your study. If you are not sure if a list item applies to your research, read the appropriate section before selecting a response.

Materials & experimental systems

n/a	Involved in the study
☐	☒ Antibodies
☐	☒ Eukaryotic cell lines
☒	☐ Palaeontology and archaeology
☒	☐ Animals and other organisms
☒	☐ Clinical data
☒	☐ Dual use research of concern

Methods

n/a	Involved in the study
☐	☒ ChIP-seq
☒	☐ Flow cytometry
☒	☐ MRI-based neuroimaging

Antibodies

Antibodies used

Following antibodies were used in the study that are detailed in the methods section.
PSIP1-P75 (Bethyl laboratory, A300-848A; 1:1000 dilution for Immunobloting; 1:250 for IF),
PSIP1-P75/P52 (Abcam, ab177159; 1:1000 dilution for Immunobloting),
RNA-DNA hybrid clone S9.6 (EMD Millipore, MABE 1095; 1:1000 dilution for slotblotting; 1:250 for IF),
PCNA (Santa Cruz Biotechnology, sc-9857-R; 1:200 dilution for Immunobloting),
RNA Pol II (8WG16) (Covance/Biolegend, MMS-126R; 1:250 for IF),
γ-H2AX (Abcam, AB81299; 1:1500 dilution for Immunobloting; 1:250 for IF),
Anti-mouse IgG-HRP (Santa Cruz Biotechnology, SC2031; 1:1500 dilution for Immunoblotting),
β-actin (Abcam, Ab8226), PARP1 (Abcam, Ab191217; 1:1500 dilution for Immunobloting),
53BP1 (Abcam, Ab175933; 1:1000 dilution for Immunobloting; 1:250 for IF),
γ-H2AX (Abcam, Ab25350; 1:1000 dilution for Immunobloting; 1:250 for IF),
α-rabbit IgG (Alexa Fluor®594; Abcam, Ab150080; 1:750 dilution for IF),
α-rabbit IgG (Alexa Fluor®488; Abcam, Ab150077; 1:750 dilution for IF),
α-mouse IgG (Alexa Fluor®488; Abcam, Ab150077; 1:750 dilution for IF)

Validation

Knockout validations were performed for PSIP1 antibodies (Pradeepa et al PloS Genetics 2012), other antibodies are previously used in publications
For other antibodies validation details can be found in the company websites

Eukaryotic cell lines

Policy information about cell lines and Sex and Gender in Research

Cell line source(s)

RWPE1, LNCaP and PC3 cell lines are procured from ATCC, HEK293T cells were sourced from Diego Villar lab (Blizard Institute)

Authentication

Cell lines are tested mycoplasma free,

Mycoplasma contamination

Cells were routinely tested for mycoplasma contamination

Commonly misidentified lines
(See ICLAC register)

No commonly misidentified cell lines used in the study

ChIP-seq

Data deposition

☒ Confirm that both raw and final processed data have been deposited in a public database such as GEO.

☒ Confirm that you have deposited or provided access to graph files (e.g. BED files) for the called peaks.

Data access links
May remain private before publication.

All NGS data are available under GSE220234
CUT&Tag-Seq data GSE220231:
RNA-Seq data GSE220232:
TT-Seq data GSE220233:
HEK293T CUT&Tag data: GSE245788

Files in database submission

GSM6797045 RWPE1, Contol-sh IgG Rep1
GSM6797046 RWPE1, Contol-sh IgG Rep2
GSM6797047 RWPE1, PSIP1-sh IgG Rep1
GSM6797048 RWPE1, PSIP1-sh IgG Rep2
GSM6797049 RWPE1, Contol-sh PSIP1 Rep1

GSM6797050 RWPE1, Contol-sh PSIP1 Rep2
GSM6797051 RWPE1, PSIP1-sh PSIP1 Rep1
GSM6797052 RWPE1, PSIP1-sh PSIP1 Rep2
GSM6797053 RWPE1, Contol-sh S9.6 Rep1
GSM6797054 RWPE1, Contol-sh S9.6 Rep2
GSM6797055 RWPE1, PSIP1-sh S9.6 Rep1
GSM6797056 RWPE1, PSIP1-sh S9.6 Rep2
GSM6797057 RWPE1, Contol-sh YH2AX Rep1
GSM6797058 RWPE1, Contol-sh YH2AX Rep2
GSM6797059 RWPE1, PSIP1-sh YH2AX Rep1
GSM6797060 RWPE1, PSIP1-sh YH2AX Rep2
GSM6797061 RWPE, Control-sh RNAseq Rep1
GSM6797062 RWPE, Control-sh RNAseq Rep2
GSM6797063 RWPE, Control-sh RNAseq Rep3
GSM6797064 RWPE, PSIP1-sh RNAseq Rep1
GSM6797065 RWPE, PSIP1-sh RNAseq Rep2
GSM6797066 RWPE, PSIP1-sh RNAseq Rep3
GSM6797067 RWPE, Control-sh TT-seq Rep1
GSM6797068 RWPE, Control-sh TT-seq Rep2
GSM6797069 RWPE, PSIP1-sh TT-seq Rep1
GSM6797070 RWPE, PSIP1-sh TT-seq Rep2
GSM7849022 HEK293T, PSIP1-sh RNASEH YH2AX Rep1
GSM7849023 HEK293T, PSIP1-sh RNASEH IgG Rep1
GSM7849024 HEK293T, PSIP1-sh RNASEH IgG Rep2
GSM7849025 HEK293T, PSIP1-sh RNASEH PSIP1 Rep1
GSM7849026 HEK293T, PSIP1-sh RNASEH PSIP1 Rep2
GSM7849027 HEK293T, PSIP1-sh RNASEH S96 Rep1
GSM7849028 HEK293T, PSIP1-sh RNASEH S96 Rep2
GSM7849029 HEK293T, PSIP1-sh control-plasmid YH2AX Rep1
GSM7849030 HEK293T, PSIP1-sh control-plasmid YH2AX Rep2
GSM7849031 HEK293T, PSIP1-sh control-plasmid IgG Rep1
GSM7849032 HEK293T, PSIP1-sh control-plasmid IgG Rep2
GSM7849033 HEK293T, PSIP1-sh control-plasmid PSIP1 Rep1
GSM7849034 HEK293T, PSIP1-sh control-plasmid PSIP1 Rep2
GSM7849035 HEK293T, PSIP1-sh control-plasmid S96 Rep1
GSM7849036 HEK293T, PSIP1-sh control-plasmid S96 Rep2
GSM7849037 HEK293T, Control-sh RNASEH YH2AX Rep1
GSM7849038 HEK293T, Control-sh RNASEH YH2AX Rep2
GSM7849039 HEK293T, Control-sh RNASEH IgG Rep1
GSM7849040 HEK293T, Control-sh RNASEH IgG Rep2
GSM7849041 HEK293T, Control-sh RNASEH PSIP1 Rep1
GSM7849042 HEK293T, Control-sh RNASEH PSIP1 Rep2
GSM7849043 HEK293T, Control-sh RNASEH S96 Rep1
GSM7849044 HEK293T, Control-sh RNASEH S96 Rep2
GSM7849045 HEK293T, Control-sh control-plasmid YH2AX Rep1
GSM7849046 HEK293T, Control-sh control-plasmid YH2AX Rep2
GSM7849047 HEK293T, Control-sh control-plasmid IgG Rep1
GSM7849048 HEK293T, Control-sh control-plasmid IgG Rep2
GSM7849049 HEK293T, Control-sh control-plasmid PSIP1 Rep1
GSM7849050 HEK293T, Control-sh control-plasmid PSIP1 Rep2
GSM7849051 HEK293T, Control-sh control-plasmid S96 Rep1
GSM7849052 HEK293T, Control-sh control-plasmid S96 Rep2

Genome browser session
(e.g. UCSC)

Provide a link to an anonymized genome browser session for "Initial submission" and "Revised version" documents only, to enable peer review. Write "no longer applicable" for "Final submission" documents.

Methodology

Replicates

Two biological replicates were used for analysis

Sequencing depth

we have used minimum of 2 million reads recomended for CUT&Tag

Antibodies

Detailed in the methods

Peak calling parameters

Peaks on the mapped reads were called using the SEACR tool using IgG as a background with “norm and relaxed” options. For each replicate per sample, the peaks were called using both individual IgG replicates as background. The peaks obtained were pooled, only the peaks consistent in both replicates were retained, and coordinates were merged. These peaks were used for further analyses. Peaks were processed using the bedtools intersect option to filter the peaks unique to either WT (PSIP parks) or KD (YH2AX and S9.6 peaks). Peak distribution across genomic landmarks (TSS, gene body or intergenic) was done on the coordinates obtained from the UCSC genome browser using bedtools intersect.

Data quality

Describe the methods used to ensure data quality in full detail, including how many peaks are at FDR 5% and above 5-fold enrichment.

Software

The signal files for merged samples were generated using the deepTools bamCoverage option with the following parameters: --binSize 20 –normalise Using CPM --scaleFactor --smoothLength 60 --extended 150 –centerReads. The signal was normalised between WT and KD for each target by comparing the reads mapping to the E. coli genome. The bigwigs generated were used for viewing on the genome browser or plotting as a heatmap or average summary plot on the genomic landmarks and peaks. Matrices generated through computeMatrix with reference-point or scale-region option were used as input for heatmap (plotHeatmap) or average summary (plotProfile), or violin (R package ggplot2) plots. The genome browser views were captured on the UCSC genome browser.
RNAseq and TTseq
Pair-end reads for the RNA-seq, and TT-seq were aligned against the human genome (hg38) via STAR aligner following the Bluebee-CORALL pipeline of mapping. The replicates were merged for each sample using SAMtools merge, followed by filtering out the multi-mapped reads by defining MAPQ as 255. The bigwigs were generated using the deepTools bamCoverage tool with the option to normalise using RPKM. These bigwigs were used for comparing the signal in the genome browser or plotting heatmaps, average summary plots or violin plots on the genes and peaks datasets. Differential gene expression for the RNAseq was performed through the DESeq2 package. The fragments count matrix was generated using the Subreads feature counts option. This count matrix was subjected to DESeq2, and the result obtained from the analysis was used to plot as a Volcano plot.
